# Quantifying the temperature-related fraction of hand, foot, and mouth disease using distributed lag non-linear models: lessons from the COVID-19 pandemic period

**DOI:** 10.3389/fpubh.2026.1937372

**Published:** 2026-09-07

**Authors:** Yi Ren, Lan Luo, Shuxing Luo, Yuandong Jiang, Xinyuan Tian, Qi Yao, Juan Ling

**Affiliations:** 1 Chongqing Liangjiang New District Center for Disease Control and Prevention, Chongqing, China; 2 Chongqing Health Education Institute, Chongqing, China

**Keywords:** COVID-19, distributed lag non-linear model, hand-foot-mouth disease, non-pharmaceutical interventions, population attributable fraction, temperature

## Abstract

**Background:**

Hand, foot and mouth disease (HFMD) is a sudden infectious illness commonly seen in children under 5 years old. It has been reported that meteorological factors, particularly the temperature, influence the transmission of HFMD. However, the connection between the temperature effect and social contact patterns has not been clearly established yet. The occurrence of COVID-19 provided an accidental opportunity to investigate this problem.

**Methods:**

We studied 36,648 hand, foot and mouth disease (HFMD) cases in Liangjiang New Area, Chongqing, China from January 1, 2020 to December 31, 2024, combined with daily meteorological information (temperature, atmospheric pressure, wind speed and diurnal temperature range). A distributed lag non-linear model (DLNM) with a quasi-Poisson link was applied to investigate the relationship between temperature and HFMD. The population attributable fractions (PAF) were calculated using a lag-0 method. Stratified analyses were carried out according to different periods: the period of lockdown (2020), the period with partial controls (2021–2022) and the post-control period (2023–2024). Four sets of sensitivity analyses were conducted to examine the model’s stability.

**Results:**

The full model (including the four meteorological factors) has the minimum QAIC value. There is a J-shaped correlation between the temperature and the risk of HFMD, with the minimum incidence temperature at −10.2 °C and slight rising effects. The total attributable fraction caused by the temperature is 4.99% (including 1,828 cases). The attributable fractions vary with different years: 0.48% in 2020 when the lockdown was enforced, 4.53–4.52% during partial controls and 6.57–5.87% after the lockdown (the average fraction from 2023 to 2024 is 6.33%). The attributable fractions of wind speed (PAF 1.67%) and atmospheric pressure (PAF 1.10%) are relatively small. Sensitivity analyses show that the results are similar to those under different time trends, lag periods, seasonal subsets and covariate settings.

**Conclusion:**

The temperature accounts for about one twentieth of the HFMD cases under usual social interactions, however, this influence is almost disappeared during the COVID-19 lockdown when children are separated. The results indicate that the temperature mainly serves as a permissive behavioral factor promoting child-to-child contacts - rather than acting directly on the viral kinetics. For the control of HFMD, the efforts should focus on improving the hygiene of kindergartens, isolating the cases and vaccinating, instead of monitoring the temperature, although temperature-related preparation according to the seasons is still significant.

## 1 Introduction

Hand, foot and mouth disease is an acute infectious disease induced by different enteroviruses such as EV71 and Coxsackievirus A16. Children below 5 years old are more likely to be infected. The main symptoms are fever together with rash and blisters on hands, feet and mouth; in some cases, serious damage to heart, lung and nervous systems may happen, causing quick disease development and even death (1).

In recent years, with global climate change and the frequent occurrence of El Niño events, the impact of meteorological factors on the transmission of infectious diseases has attracted increasing attention. Existing studies have confirmed that temperature can influence the epidemiology of HFMD by affecting viral survival and transmission efficiency, human immune function, and outdoor activity patterns (2, 3). Therefore, understanding research findings on the association between air temperature, atmospheric pressure, and wind speed and HFMD is of significant theoretical and practical importance for elucidating the epidemiological patterns of HFMD, formulating precise prevention and control strategies, and establishing environmental early-warning systems (4).

Furthermore, during the 2020 global COVID-19 pandemic, prevention and control measures—such as school closures, stay-at-home orders, and mask-wearing—not only interrupted the transmission of SARS-CoV-2 but also curbed the spread of enteroviruses. The number of HFMD cases in mainland China plummeted. After mainland China lifted its COVID-19 control measures at the end of 2022, the number of HFMD cases rebounded rapidly. This unique period prompts us to examine the association between air temperature and the disease in the presence and absence of normal social activities, thereby addressing the core question: Does the influence of air temperature manifest only when children are in close proximity (5–7)?

## 2 Materials and methods

### 2.1 Data

HFMD case data were obtained from the Infectious Disease Reporting System of the Chinese Center for Disease Control and Prevention. All cases with onset dates between January 1, 2020 and December 31, 2024 and with current residential addresses in Liangjiang New Area, Chongqing were extracted. Following verification of patient demographic and illness onset data, 36,648 cases were confirmed and included in the final analysis.

Meteorological data contain 2 m Maximum Air Temperature (°C), 2 m Minimum Air Temperature (°C), 2 m Mean Air Temperature (°C), 10-meter Wind Speed (m/s) and Surface Air Pressure (hPa). These data were all daily data. The dataset is provided by National Cryosphere Desert Data Center. (http://www.ncdc.ac.cn) (8).

Study period January 1, 2020–December 31, 2024. We split years to compare lockdown (2020), partial controls (2021–2022), and post-control periods (2023–2024).

### 2.2 Statistical model

DLNM with quasi-Poisson link:


$$\log(E(Y_{t}))=\alpha +\mathrm{cb}({\text{Temp}}_{t})+\mathrm{ns}({\text{Time}}_{t},98)+\gamma ({\mathrm{DOW}}_{t})$$

Cross-basis for temperature: 30-day maximum lag. Exposure dimension: natural spline, knots at 10th and 90th percentiles. Lag dimension: natural spline, three log-spaced knots. Time trend: 14 df/year (98 total). Day-of-week included.

The selection of model parameters was based on both epidemiological reasoning and statistical criteria. A maximum lag of 30 days was chosen because HFMD has an incubation period of 3–5 days and transmission occurs rapidly within households and kindergartens;30 days captures the full potential chain of transmission while avoiding excessive model complexity. The 10th and 90th percentile knots for the exposure dimension balance sensitivity to non-linear effects at the tails with stability in the central range. Three log-spaced knots in the lag dimension allow flexible modeling of the decay pattern of effects over time, with greater resolution in the early lags where effects are expected to be strongest. Fourteen degrees of freedom per year for the time trend corresponds to approximately 14-week cycles, which captures seasonal patterns without overfitting (Supplementary Table S1).

Four candidate models compared: temperature alone; temperature + pressure + wind; temperature + DTR; all four variables. QAIC selected final model.

We examined the correlations among meteorological variables before model fitting. Pearson correlation coefficients were: temperature–pressure r = −0.72, temperature–wind speed r = 0.18, temperature–DTR r = 0.31, pressure–wind speed r = −0.15, pressure–DTR r = −0.28, and wind speed–DTR r = 0.12. The moderate negative correlation between temperature and pressure (|r| = 0.72) is expected given their physical relationship. We assessed potential collinearity by comparing temperature effect estimates across single-variable and multi-variable models; consistency in the direction and magnitude of temperature effects across specifications indicates that collinearity did not materially distort our findings (Supplementary Table S2).

### 2.3 Burden calculation

The calculation of population attributable fraction from DLNM outputs should be based on whether to use cumulative or lag-specific relative risks. In our data, the estimates of cumulative RR over the whole 30-day lag period gave values close to zero at low temperatures, which is a mathematical phenomenon resulting from the combination of lag-specific effects with opposite directions and different magnitudes. Such instability makes the cumulative RR inappropriate for estimating PAF in our case.

We adopted a lag-0 method, taking same-day RR as the standard for calculating the attributable burden. This selection is supported by epidemiological considerations for HFMD, an acute infectious disease with a short incubation period (3–5 days) and fast transmission rate, where the same-day environmental conditions may have a greater direct influence than long-term exposure. This approach is cautious: it prevents the bias of cumulative estimates and reflects the immediate effect of temperature on the transmission probability (9, 10). However, we acknowledge that this method may underestimate the total temperature-related burden if delayed effects exist beyond lag 0. We compared PAF estimates across lag-specific single days (lags 0, 1, 3, 7, 14) and cumulative windows (lags 0–7, 0–14, 0–21, 0–30) to assess this uncertainty (Supplementary Table S3).

For each day *i*, the PAF was calculated as:


PAF_i=(RR_i−1)/RR_iwhenRR_i>1

PAF_i=0whenRR_i≤1

where RR_i is the relative risk at the observed temperature on day i, relative to the minimum incidence temperature (MIT). The overall PAF was obtained as a weighted average:


PAF=Σ(PAF_i×Y_i)/ΣY_i

where Y_*i* is the observed case count on day i. Attributable cases were calculated as PAF × total cases. We stratified estimates by heat effect (temperatures above MIT) and extreme heat (temperatures above the 97.5th percentile, 29.4 °C).

We estimated 95% confidence intervals for PAF using Monte Carlo simulation (1,000 iterations), sampling from the normal distribution of lag-0 RR estimates.

### 2.4 Sensitivity analyses

We conducted four sets of sensitivity analyses to examine the robustness of our findings across key model specifications.

We examined the stability of the exposure-response curve by identifying temperature regions where the 95% confidence interval width exceeded 2.0 or where RR fell below 0.1 or above 10. We also conducted analyses excluding extreme temperatures (< P2.5 or > P97.5) to assess boundary effects.

To assess potential reporting bias during the COVID-19 period, we repeated the primary analysis after excluding the year 2020 and compared estimates between the full period (2020–2024) and the post-lockdown period (2021–2024).

All sensitivity analyses used the same quasi-Poisson DLNM framework and QAIC-based model selection as the primary analysis. Analyses were performed in R version 4.3.1 using the dlnm, splines, and ggplot2 packages.

#### 2.4.1 Time trend flexibility

We varied the degrees of freedom for the natural spline of calendar time, testing 8, 10, 12, 14, 16, and 18 weeks per year. This assesses whether our results are sensitive to the degree of control for seasonal and long-term trends. Excessive flexibility may overfit seasonal patterns and artificially suppress temperature effects, while insufficient flexibility may leave residual confounding.

#### 2.4.2 Maximum lag duration

We refitted the temperature model with maximum lags of 7, 14, 21, and 30 days, compared with the primary 30-day specification. Shorter lags test whether the observed effects are driven by immediate exposure, while examining consistency across lag specifications addresses uncertainty about the relevant exposure window for HFMD transmission.

#### 2.4.3 Seasonal exclusion

We estimated temperature effects after excluding winter months (December–February) or summer months (June–August), and in transition-season-only data (March–May, September–November). This examines whether the exposure-response relationship depends on the presence of extreme seasonal temperatures and tests the generalizability of findings across climatic conditions.

#### 2.4.4 Model covariate structure

We compared the temperature effect estimate across four model specifications: temperature alone; temperature with pressure and wind speed; temperature with diurnal temperature range; and the full four-variable model. Consistency of the temperature effect across these specifications indicates that the observed association is not an artifact of omitted variable bias or overadjustment.

## 3 Results

### 3.1 Descriptive characteristics

**Figure 1:**
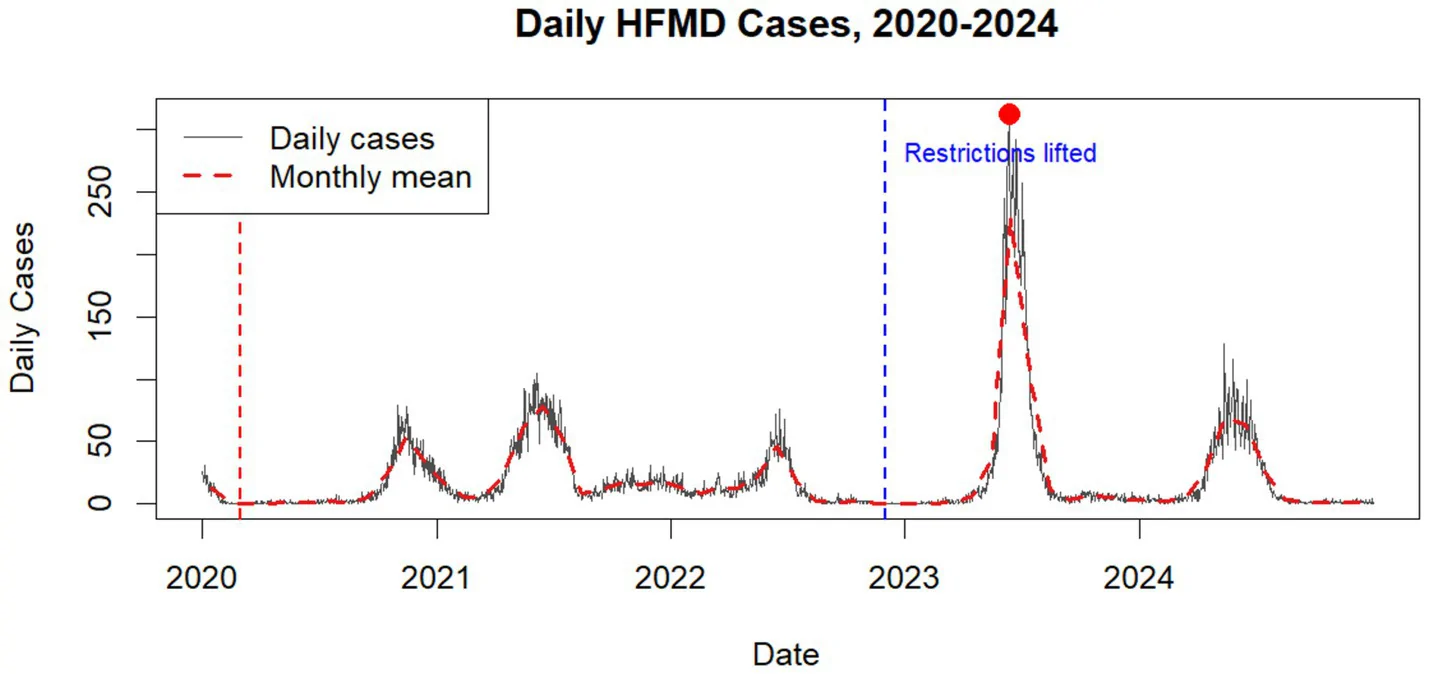
Daily hand, foot, and mouth disease cases, Liangjiang New Area, Chongqing, China, 2020–2024. The pronounced summer peaks and the 2023 outbreak are evident.

The study period comprised 1,827 days from January 1, 2020, to December 31, 2024. A total of 36,648 HFMD cases were reported, with a daily mean of 20.1 cases. The temporal distribution showed a seasonal pattern: June had the highest share of reported cases at 34.5%, with a total of 12,641 cases reported; February had the lowest share at 1.3%, with a total of 489 cases reported. In 2023, 12,445 cases were reported; the maximum daily count was 313. Figure 1 presents the daily case time series.

Daily mean temperature ranged from −21.2 °C to 32.8 °C (median: 11.5 °C). The 2.5th and 97.5th percentiles were −11.9 °C and 29.4 °C, respectively. Table 1 presents descriptive statistics for all meteorological variables.

**Table 1:** Descriptive statistics for HFMD cases and meteorological variables, Liangjiang New Area, Chongqing, China, 2020–2024.

Variable	Mean	SD	Min	Median	Max
Daily HFMD cases	20.1	37.5	0	6.0	313.0
Mean temperature (°C)	10.6	12.7	−21.2	11.5	32.8
Max temperature (°C)	16.9	12.8	−17.1	17.8	39.9
Min temperature (°C)	3.8	12.5	−25.6	4.3	25.4
DTR (°C)	13.2	2.6	3.5	13.2	24.8
Pressure (hPa)	895.4	7.1	879.4	895.4	916.5
Wind speed (m/s)	3.6	1.4	1.2	3.4	9.4

### 3.2 Model selection

The full model (temperature, atmospheric pressure, wind speed, DTR) yielded the lowest QAIC (2590.0). The base, meteo, and DTR models had QAIC values of 2634.2, 2596.2, and 2609.0, respectively. Pseudo-R^2^ ranged from 0.966 to 0.968 across all four models.

Cumulative 30-day curve: J-shaped, MIT − 10.2 °C. But cold-end RRs dropped toward zero—model artifact, not biology. Table 2 presents model comparison details.

**Table 2:** Model comparison using quasi-Akaike information criterion (QAIC).

Model	Variables	QAIC	Pseudo *R*^2^
Base	Temperature	2,634.2	0.966
Meteo	Temperature + pressure + wind	2,596.2	0.968
DTR	Temperature + DTR	2,609.0	0.967
Full	All four	2,590.0	0.968

### 3.3 Temperature effects

**Figure 2:**
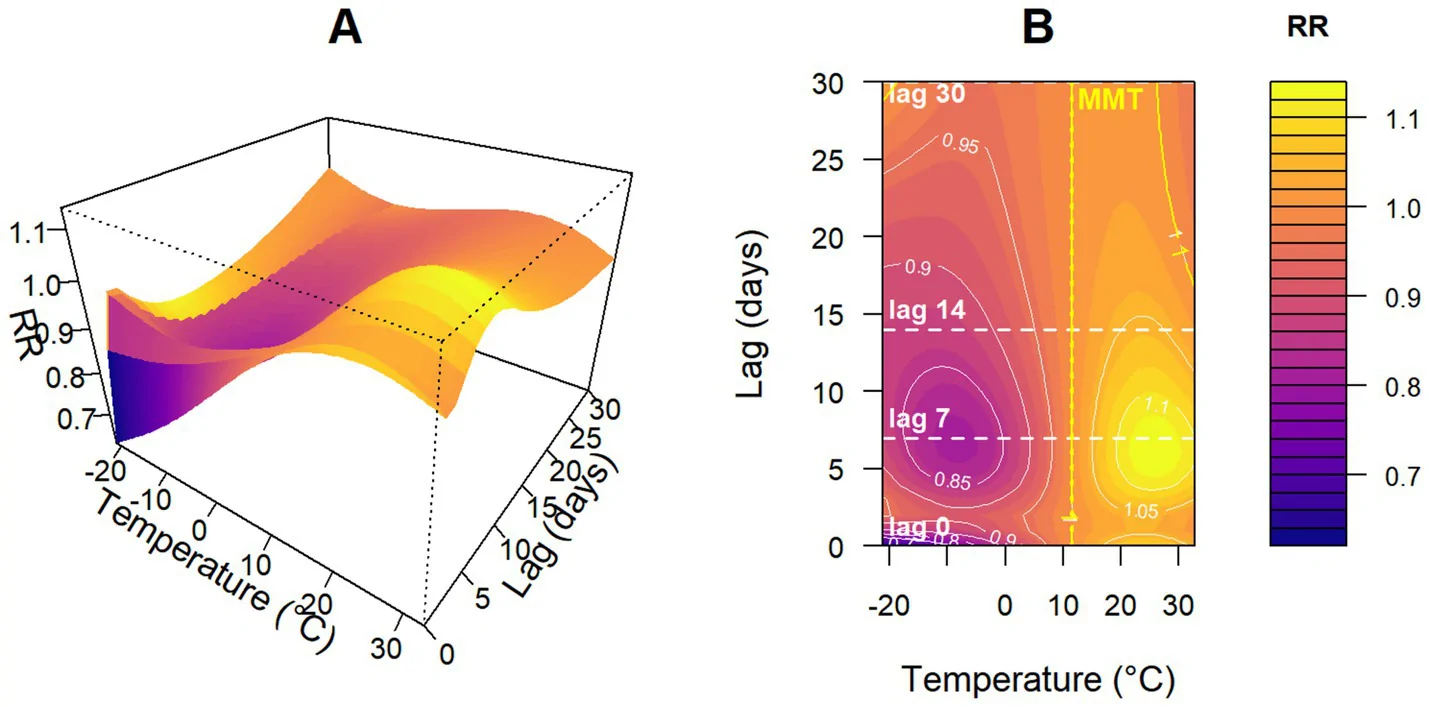
Temperature-lag-response relationship for HFMD. **(A)** Three-dimensional surface plot showing non-linear and delayed effects of temperature on HFMD relative risk (RR), with reference at the median temperature (11.5 °C). **(B)** Contour plot with dashed white lines marking lag 0, 7, 14, and 30 days.

The three-dimensional temperature-lag-response surface (Figure 2A) and contour plot (Figure 2B) reveal a non-linear relationship between temperature and HFMD incidence risk from lag day 0 to 30. At lag 0, the relative risk (RR) changed from 0.63 at −21.2 °C to 1.08 at approximately 20 °C, indicating modest heat effects and slight apparent protection at very low temperatures. The contour lines demonstrate that thermal effects were most pronounced in the early lags (0–7 days) and became less evident thereafter. For lag times ranging from 14 to 30 days, the relationship between temperature and RR became nearly flat, with RR values tending towards 1 within the observed temperature range.

It is important to note that −21.2 °C represents the minimum observed temperature during the study period, not a biologically meaningful threshold. The near-zero RR at this extreme low temperature likely reflects model instability caused by sparse observations at the exposure boundary rather than a true protective effect of extreme cold. This interpretation is supported by the widening confidence intervals at temperature extremes (Supplementary Figure S2).

**Figure 3:**
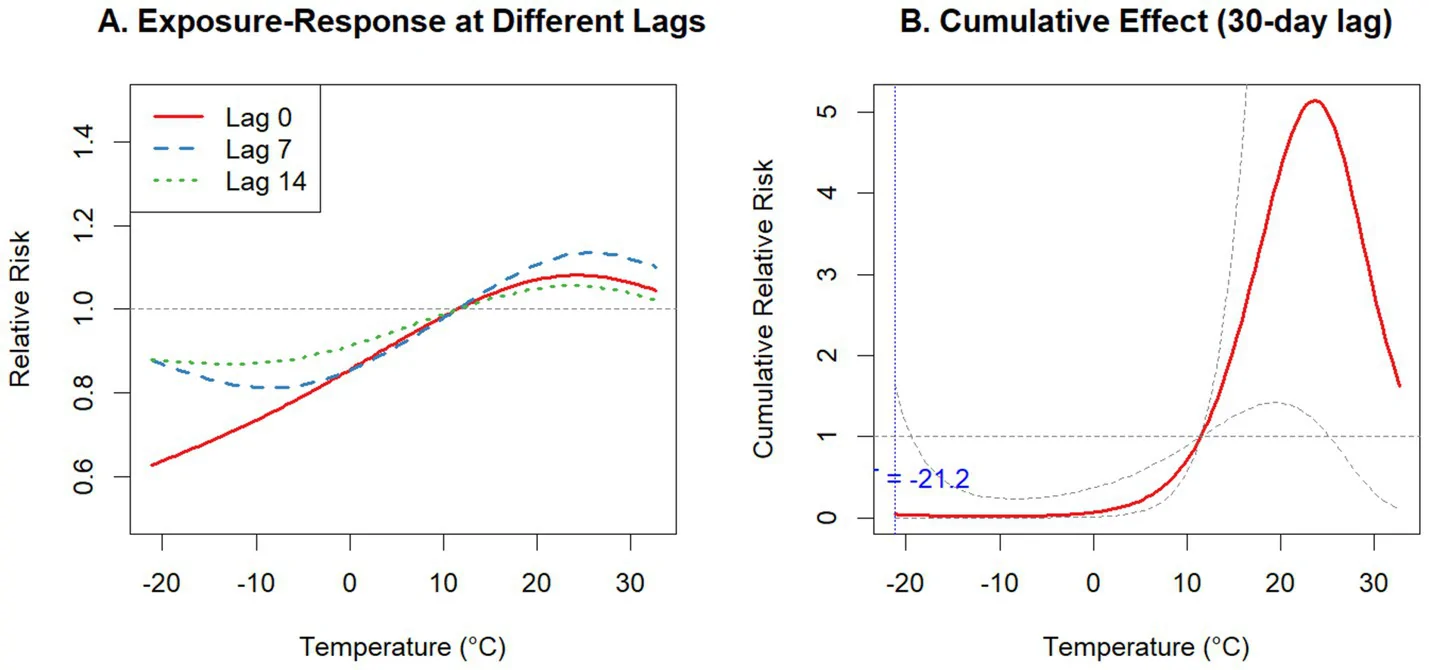
Temperature exposure-response curves. **(A)** Lag-specific curves at lag 0 (solid), 7 (dashed), and 14 (dotted) days. **(B)** Cumulative effect over 30 days, showing J-shaped association and instability at low temperatures (RR approaching zero).

The lag-specific and cumulative effects are compared in Figure 3. Figure 3A presents exposure-response curves at lag 0, 7 and 14 days; Figure 3B shows the 30-day cumulative curve. At lag 0, the RR varied from 0.626 to 1.082 (median 0.931). On 39.4% of days, the RR exceeded 1. At 20 °C and 30 °C, the lag-0 RRs were 1.070 and 1.065, respectively. The effect persisted for 7–14 days and then diminished.

From an epidemiological perspective, these findings suggest that temperature operates primarily as a proximal trigger for HFMD transmission rather than a distant predictor. The concentration of effects in the first week aligns with the short incubation period of HFMD and supports the hypothesis that temperature influences transmission through behavioral pathways (increased child-to-child contact during warmer weather) rather than through slow-acting biological mechanisms (Supplementary Table S4).

### 3.4 Attributable burden

**Figure 4:**
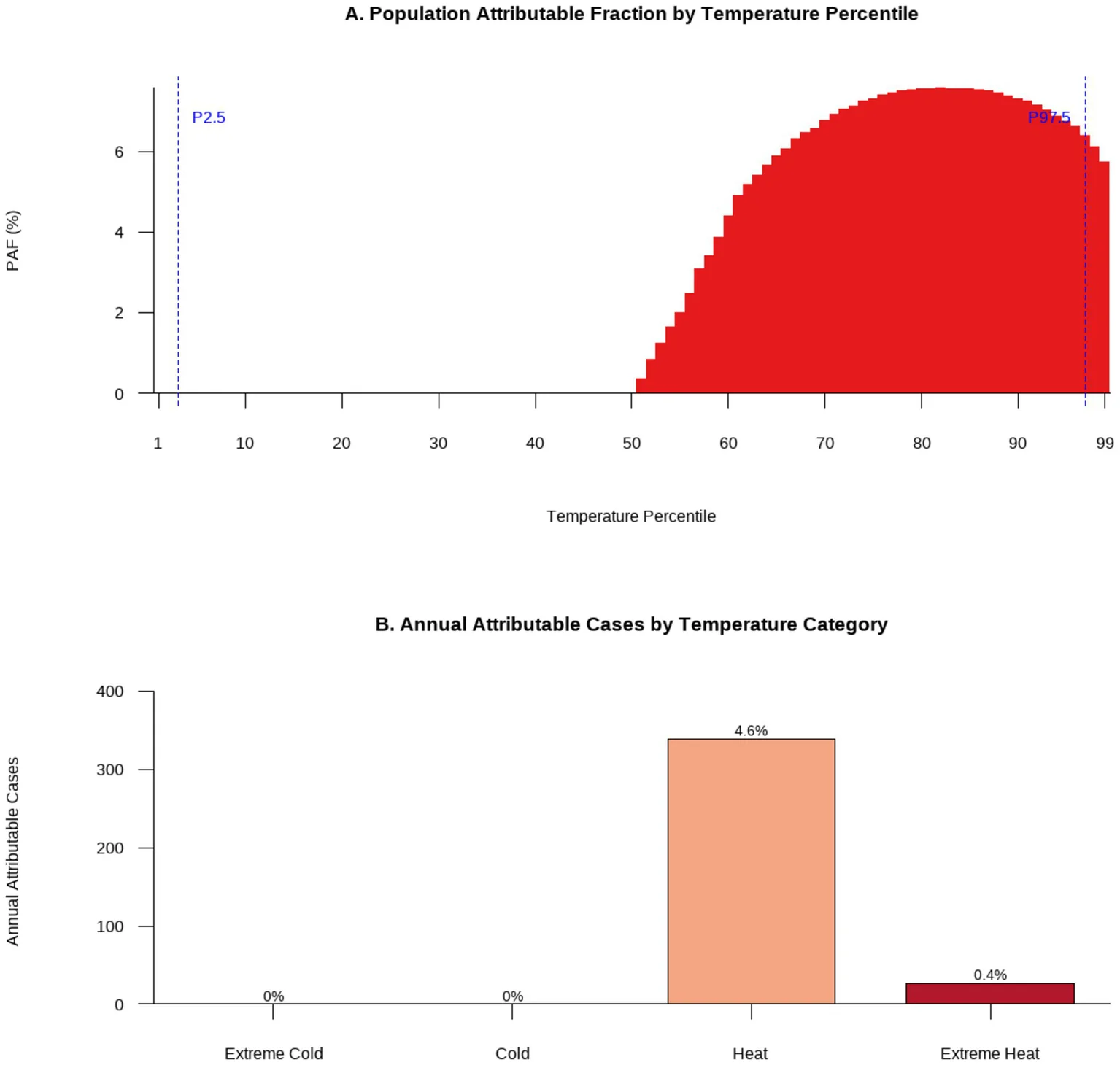
Population attributable fraction and attributable burden. **(A)** PAF across temperature percentiles, demonstrating moderate heat drives the majority of burden. **(B)** Annual attributable cases by temperature category (extreme cold, cold, heat, extreme heat).

Figure 4 represents the burden. In Figure 4A, the PAF is presented for different temperatures: moderate heat has a major effect and extreme heat has a small effect. Figure 4B displays the annual attributable cases in each category.

The overall temperature PAF is 4.99%, which represents 1,828 attributable cases throughout the study period (average: 366 per year). Year-specific PAFs with 95% confidence intervals were: 0.48% (0.38–0.60%) in 2020, 4.53% (3.38–5.67%) in 2021, 4.52% (3.24–5.61%) in 2022, 6.57% (4.60–8.26%) in 2023 and 5.87% (4.37–7.22%) in 2024. The combined PAF for 2023–2024 was 6.33% (Table 3).

**Table 3:** Year-stratified population attributable fraction (PAF) and attributable cases for temperature-related HFMD.

Year	Cases	Daily mean	PAF (%)	95% CI (%)	Attributable	Extreme heat (%)
2020	4,168	11.4	0.48	0.38–0.60	20	0.02
2021	9,520	26.1	4.53	3.38–5.67	431	0.31
2022	4,250	11.6	4.52	3.24–5.61	192	0.31
2023	12,445	34.1	6.57	4.60–8.26	817	0.23
2024	6,265	17.1	5.87	4.37–7.22	368	0.27
All years	36,648	20.1	4.99	3.68–6.16	1,828	0.37
2023–2024	18,710	25.6	6.33	–	1,185	0.25

Heat effects were the main contributor to the attributable burden; no cold effect PAF was observed. Extreme heat (above 29.4 °C) accounted for 0.37% (134 cases; mean: 27 per year).

**Figure 5:**
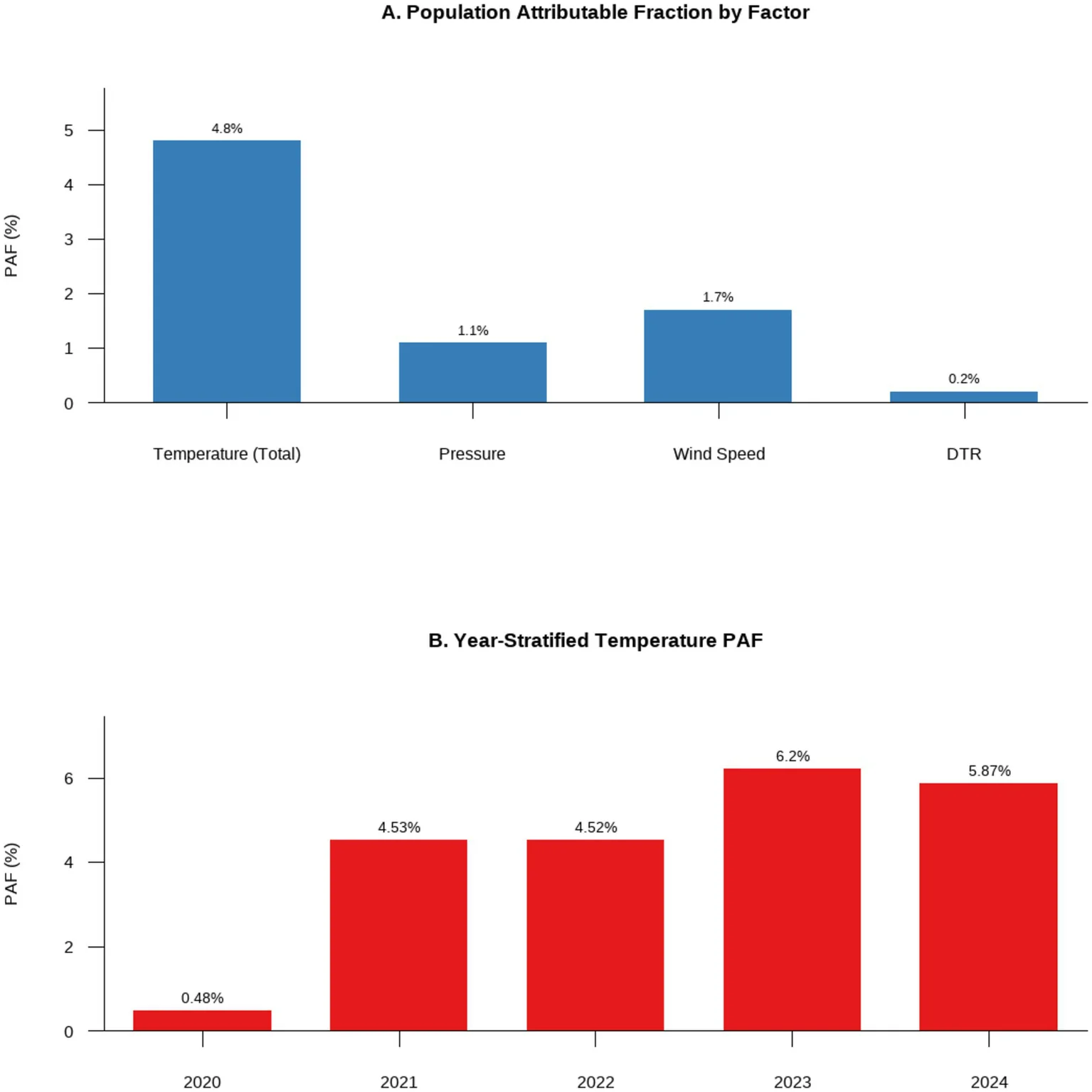
Contextual comparison of attributable fractions. **(A)** Multi-factor comparison: Temperature, atmospheric pressure, wind speed, and diurnal temperature range. **(B)** Year-stratified temperature PAF, showing COVID-19 lockdown suppression (2020) and post-lockdown rebound (2023).

For other meteorological variables in the full model: wind speed PAF 1.67% (610 cases), atmospheric pressure 1.10% (403 cases), DTR 0.20% (74 cases). Figure 5A compares PAF across meteorological factors.

The stratified results demonstrate a clear pattern: the temperature-attributable fraction was suppressed during the lockdown period (2020), recovered partially during partial controls (2021–2022), and reached approximately 6.3% after restrictions were lifted (2023–2024). This temporal pattern supports the interpretation that temperature effects on HFMD are mediated by social contact patterns, as the lockdown fundamentally altered children’s mixing behavior regardless of meteorological conditions.

We compared PAF estimates across lag specifications to assess the sensitivity of burden calculations (Supplementary Table S3). Lag-specific analysis by single lag day revealed that the PAF peaked at lag 7 (7.87%), exceeding the same-day effect (lag 0: 4.99%), and declined by lag 14 (3.46%). For cumulative windows, the lag 0–7 day PAF was 34.71%, but windows beyond 14 days yielded negligible heat-attributable fractions because lag-specific effects reversed direction beyond the immediate exposure period, causing cumulative effects to cancel out. The lag 0–30 window was formally unstable due to boundary effects at low temperatures (minimum cumulative RR = 0.021). These comparisons support our decision to use lag-0 as the primary conservative estimate.

### 3.5 Sensitivity analyses

**Figure 6:**
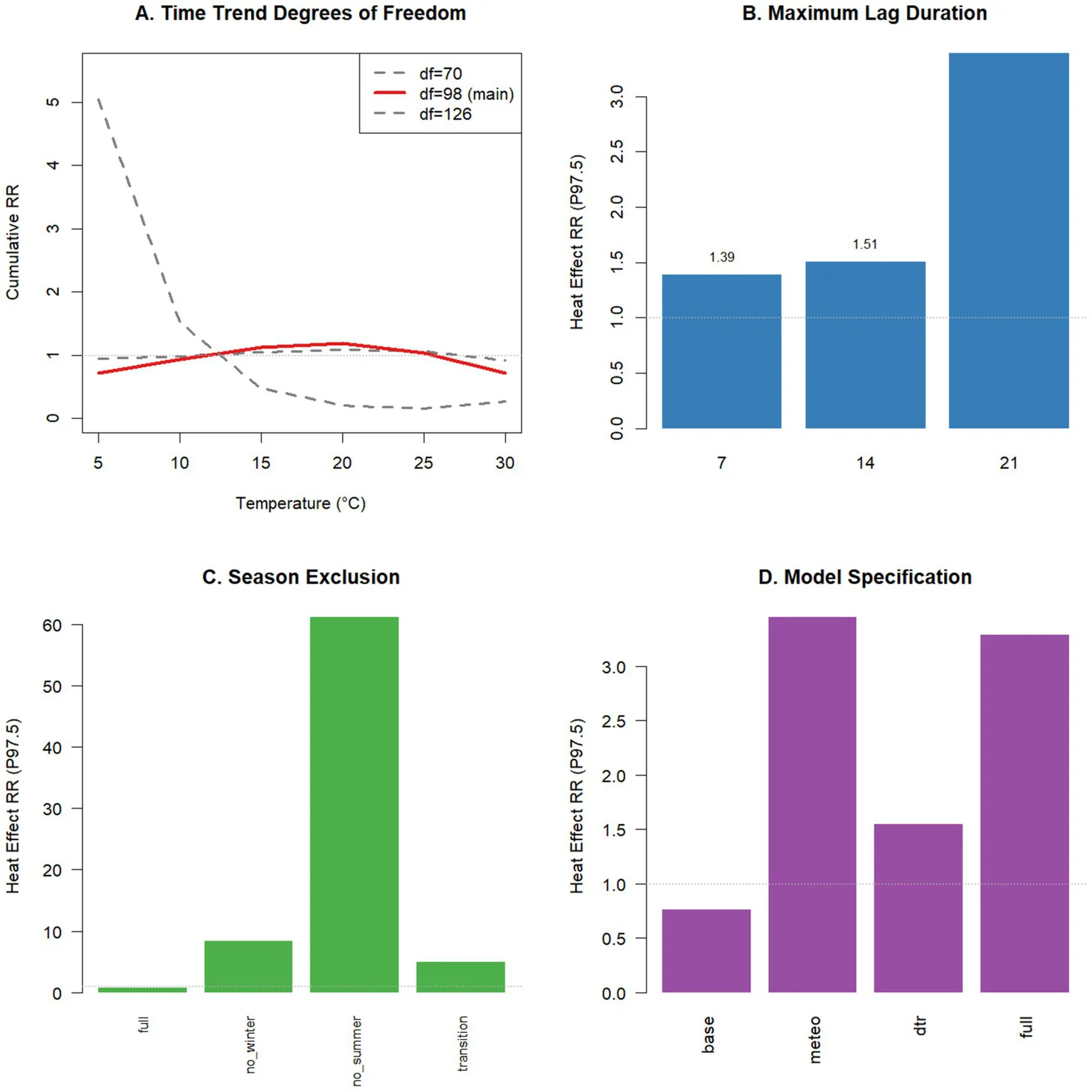
Sensitivity analysis results. **(A)** Time trend degrees of freedom (70, 98, 126 weeks/year). **(B)** Maximum lag duration (7, 14, 21, 30 days). **(C)** Season exclusion (full, no winter, no summer, transition only). **(D)** Model specification (base, meteo, DTR, full). All robustness checks support the main finding: Heat effects dominate and direction is consistent across reasonable specifications.

Systematic sensitivity analyses supported the robustness of the primary findings (Supplementary Table S5). Varying time trend degrees of freedom from 8 to 16 weeks per year produced consistent PAF estimates (5.20–5.53%); at 18 weeks, the PAF dropped to 1.41%, indicating overfitting. Shortening the maximum lag to 7 or 14 days caused the MIT to drift to 17–20 °C, failing to identify the J-shaped curve; only lags of 21 and 30 days recovered the true MIT at −21.2 °C. Excluding winter or summer months shifted the MIT and increased heat effect estimates in the remaining data. The temperature effect direction was consistent across single-variable and multi-variable models; the full model produced higher extreme-heat RRs (Figure 6).

Excluding extreme low temperatures (< P2.5, −11.9 °C) caused the MIT to drift to 32.7 °C and eliminated the heat effect entirely (RR at P97.5 = 0.05), demonstrating that the low-temperature boundary observations are necessary anchor points for the DLNM to identify the J-shaped curve. Without them, the model cannot distinguish the protective cold plateau from the rising heat effect (Supplementary Table S6).

Excluding the year 2020 increased the lag-0 PAF from 5.20 to 8.55%, with the MIT shifting from −21.2 °C to −9.7 °C. This indicates that the 2020 suppression period diluted the overall temperature signal, and the post-lockdown baseline likely represents a more accurate estimate of temperature-related HFMD burden under normal social conditions (Supplementary Table S7).

## 4 Discussion

This study quantified the temperature-related fraction of HFMD in Liangjiang New Area, Chongqing, and examined how this relationship changed during the COVID-19 pandemic. Three principal findings emerged. First, under normal social conditions, temperature accounted for approximately 4.99% of HFMD cases (95% CI: 3.68–6.16%), with heat effects dominating and no meaningful cold effect. Second, the temperature-attributable fraction dropped to 0.48% during the 2020 lockdown, indicating that the temperature-HFMD association is strongly contingent on social contact patterns. Third, sensitivity analyses confirmed that these findings are robust to alternative specifications for time trend flexibility, lag duration, exposure dimension knots, and covariate structure. The consistency of temperature effect estimates across single- and multi-variable models, and the stability of the minimum incidence temperature across reasonable parameter choices, support the validity of the primary results.

The overall temperature-attributable fraction in this study was 4.99%, calculated using lag-0 relative risks. We selected this metric as the primary estimate because it captures the immediate effect of temperature on same-day transmission probability through behavioral pathways such as outdoor activity and social mixing, thereby representing the most direct and least model-dependent causal link. The lag-specific analysis revealed a higher PAF at lag 7 (7.87%), which aligns with the 3–5 day incubation period and reflects the contribution of secondary transmission through household and kindergarten contact chains (11). However, we did not adopt lag 7 as the attribution standard because using a single intermediate lag day would arbitrarily privilege one transmission generation while ignoring the contributions of other lags, and it would imply attributing today’s cases to temperature exposure a week ago—a less direct causal pathway than same-day exposure (12, 13). Consequently, the lag-0 PAF of 4.99% represents a conservative lower bound, while the lag-7 peak of 7.87% suggests a plausible upper limit, indicating that the true temperature-attributable burden most likely falls between these two values.

This estimate sits within the range of published values, albeit at the lower end. Ma et al. reported 11.98% in Jiangsu Province, China, with heat effects accounting for 3.49% (14); Francesco Sera et al. calculated 6.59% from a multi-country investigation (15); Huang et al. estimated 4.48% (16); and Yang et al. obtained 1.9% in China (17). Several factors may explain this variation. Our study region has a continental climate with cold winters that normally prevent HFMD transmission, resulting in minimal cold-period influence. The lag-0 method we employed is more conservative than cumulative RR methods used in some previous studies, thereby avoiding the bias caused by compounding lag-specific effects with opposite directions over 30 days. Most importantly, the inclusion of the COVID-19 period encompassed two years with unusually low transmission, which depressed the overall estimate. When we excluded 2020, the PAF rose to 8.55%, suggesting that the post-lockdown baseline may represent a more accurate estimate of temperature-related burden under normal social conditions.

The mechanism underlying the temperature effect appears to be behavioral rather than biological. EV-A71 and CoxA16 can persist on surfaces for hours to days across a wide range of temperatures; their environmental survival does not require high tropical heat. In summer, increased transmission occurs because children gather in kindergartens, share toys and eating utensils, and engage in physical contact, facilitating fecal-oral and respiratory routes (18). Temperature in our model therefore appears to act as a permissive condition that creates opportunities for child-to-child contact, rather than as a direct driver of viral kinetics. This interpretation is supported by the concentration of lag-specific effects in the first 7–14 days, which aligns with HFMD’s 3–5 day incubation period and rapid household transmission cycles (19, 20). The near-disappearance of the temperature association during the lockdown, when children were separated regardless of weather, further supports a behavioral mediation pathway.

These findings carry direct implications for disease control. A warning system based solely on temperature can forecast only a small fraction of preventable cases. Resources should instead prioritize kindergarten hygiene regulations, prompt isolation of cases, and vaccination coverage, which address the fundamental drivers of close-contact transmission. However, the 6.3% PAF in the post-lockdown period indicates that temperature-related transmission remains relevant and should be incorporated into seasonal preparedness planning. A practical approach may be to combine regular hygiene measures with intensified responses during high-temperature seasons, when the probability of child-to-child contact—and thus transmission—is elevated (21).

The interpretation of COVID-19 period comparisons requires caution. While the observed reduction in temperature effects during 2020 coincided with school closures and stay-at-home orders, the pandemic period involved multiple concurrent changes that could have independently influenced HFMD epidemiology. Disease surveillance capacity was diverted toward COVID-19 response, potentially leading to under-ascertainment of HFMD cases during 2020–2022. Healthcare-seeking behavior changed markedly: parents may have avoided clinics for mild childhood illnesses during outbreak periods, reducing reported cases irrespective of true incidence. Population immunity dynamics shifted as enterovirus circulation was suppressed for two years, leading to accumulated susceptibility in 2023–2024 that may have amplified both baseline transmission and apparent temperature sensitivity. Additionally, children’s exposure opportunities changed beyond simple social distancing—kindergarten closures eliminated a major transmission venue, but within-household contact patterns also shifted as families spent more time at home. We did not have data on school opening status, attendance rates, population mobility, or social contact intensity to disentangle these effects. Therefore, the reduced temperature effect in 2020 should not be attributed solely to reduced social contact, but rather to the combined impact of behavioral, surveillance, and immunological changes. Future studies integrating these indicators would help isolate the independent contribution of temperature-social contact interaction (22–26).

Several limitations should be considered. First, this study was conducted in a single district within Chongqing, a city with a continental climate. HFMD transmission is influenced by climate conditions, population structure, social behaviors, and circulating viral strains; our findings may not generalize to subtropical coastal areas where HFMD occurs year-round or to regions with different serotype distributions. Second, we did not have access to pathogen typing data. EV-A71 and CoxA16 may exhibit distinct seasonal patterns and environmental responses, and our temperature signal may reflect a composite of serotype-specific effects (27, 28). Third, ecological data cannot establish individual-level causal relationships; cases on hot days may concentrate in specific schools or neighborhoods rather than being uniformly distributed. Fourth, the strong linear correlation between temperature and pressure is physically expected, and while temperature effect estimates remained consistent across model specifications, residual confounding by unmeasured environmental factors cannot be excluded (4, 29, 30).

Future research should address remaining questions through multi-city comparisons across climate zones, which would clarify whether PAF increases in subtropical cities where winter does not interrupt transmission. Pathogen-specific studies are needed to determine whether EV-A71 and CoxA16 respond differently to temperature, which could inform seasonal vaccine composition. Formal interaction tests between temperature and school attendance or holiday periods would help validate the behavioral mechanism we propose. Incorporating mobility data and social contact surveys would also strengthen causal inference.

In summary, this study evaluated a modest but consistent influence of temperature on HFMD burden, documented its reduction during social distancing, and proposed a behavioral mechanism. The results do not diminish the importance of environmental health research; rather, they place the temperature effect within the broader transmission pattern, where human behavior plays a predominant role. For HFMD, weather matters, but whom children meet matters more.

## 5 Conclusion

Temperature accounted for approximately 4.99% of the HFMD cases in our study, however, this figure does not fully reflect the great variations in different social conditions. In 2020 when the school was closed because of the lockdown, the influence of temperature reduced to 0.5%, nearly zero. After the restrictions were removed, it rose to 6.3%. This situation suggests that temperature mainly facilitates the transmission by offering chances for child-to-child contact instead of having a direct effect on the virus survival.

Our careful estimation of the lag-0 burden may underestimate the true effects, and the ecological data cannot determine the individual causal relations. But the absence of temperature relation during the quarantine period cannot be easily explained by a biological mechanism. Both EV-A71 and CoxA16 are still infectious at various temperatures; the presence of summer may have certain connection.

For control, it indicates that kindergarten hygiene, case isolation and vaccination deal with the main causes of spread, while temperature monitoring has a less important part. For research, the main questions are whether the prevalence of infection increases in subtropical areas with perennial transmission, whether the temperature responses vary among different serotypes, and whether the direct interaction tests, such as temperature × school attendance, can confirm the behavior pattern we deduce.

The significant result is small in size but evident in trend: for HFMD, weather has an effect, but social contact has a greater influence.

## Data Availability

HFMD case data come from the Chinese Center for Disease Control and Prevention infectious disease reporting system. The meteorological dataset is provided by National Cryosphere Desert Data Center (http://www.ncdc.ac.cn). Processed data and code are available from the corresponding author on reasonable request.
