# Supplementary material for: Quantifying the temperature-related fraction of hand, foot, and mouth disease using distributed lag non-linear models: lessons from the COVID-19 pandemic period
Source: Front Public Health. 2026 Sep 7;14:1937372. doi: 10.3389/fpubh.2026.1937372 (PMC13590558; doi:10.3389/fpubh.2026.1937372)
Supplement: Supplementary file 1 [file Data_Sheet_1.docx]

Supplementary Material

# 1 Supplementary Figures and Tables

## 1.1 Supplementary Figures

Supplementary Figure S1. Lag-response curves for temperature at 10th, 50th, and 90th percentiles (detailed view of Figure 2).

Supplementary Figure S2. Cumulative temperature effect curve with 95% confidence interval (detailed view of Figure 3, Panel B).

Supplementary Figure S3. Annual attributable cases by temperature category (detailed view of Figure 4, Panel B).

Supplementary Figure S4. Year-stratified temperature PAF comparison (detailed view of Figure 5, Panel B).

## 1.2 Supplementary Tables

Supplementary Table S1. Systematic comparison of model parameter specifications

A. Maximum lag duration

| **Max lag (days)** | **QAIC** | **ΔQAIC** | **MIT (°C)** | **RR at MIT** | **RR at 20°C (lag 0)** | **Lag-0 PAF (%)** |
| --- | --- | --- | --- | --- | --- | --- |
| 7 | 1,992.9 | 21.9 | 20.1 | 0.978 | 0.978 | 0.68 |
| 14 | 1,987.3 | 16.3 | 17.3 | 0.989 | 0.989 | 0.33 |
| 21 | 1,976.8 | 5.8 | −21.2 | 0.882 | 1.079 | 6.69 |
| 30 (main) | 1,971.0 | 0 | −21.2 | 0.864 | 1.073 | 5.20 |

B. Exposure dimension knot placement

| **Knot placement** | **Knot 1 (°C)** | **Knot 2 (°C)** | **QAIC** | **ΔQAIC** | **MIT (°C)** | **RR at 20°C** |
| --- | --- | --- | --- | --- | --- | --- |
| P10/P90 (main) | −7.4 | 26.5 | 1,971.0 | 0 | −21.2 | 1.073 |
| P5/P95 | −9.9 | 28.3 | 1,971.0 | 0 | −21.2 | 1.073 |
| Equidistant | −3.2 | 14.8 | 1,971.1 | 0.1 | −21.2 | 1.047 |

C. Time trend flexibility

| **Weeks/year** | **Total df** | **QAIC** | **ΔQAIC** | **MIT (°C)** | **Lag-0 PAF (%)** |
| --- | --- | --- | --- | --- | --- |
| 8 | 56 | 1,885.5 | 0 | −21.2 | 5.53 |
| 10 | 70 | 1,927.2 | 41.7 | −21.2 | 1.86 |
| 12 | 84 | 1,907.3 | 21.8 | −21.2 | 0.65 |
| 14 (main) | 98 | 1,971.0 | 85.5 | −21.2 | 5.20 |
| 16 | 112 | 1,980.8 | 95.3 | −21.2 | 5.31 |
| 18 | 126 | 1,993.2 | 107.7 | −21.2 | 1.41 |

Supplementary Table S2. Meteorological variable correlations and collinearity diagnostics

A. Pearson correlation matrix

|  | **Temperature** | **Pressure** | **Wind speed** | **DTR** |
| --- | --- | --- | --- | --- |
| Temperature | 1.00 | −0.83 | 0.01 | 0.13 |
| Pressure | — | 1.00 | −0.09 | −0.20 |
| Wind speed | — | — | 1.00 | −0.02 |
| DTR | — | — | — | 1.00 |

B. Variance inflation factor (linear approximation)

| **Variable** | **VIF** | **GVIF^(1/2df)** | **Assessment** |
| --- | --- | --- | --- |
| Temperature | 27.82 | 5.27 | Severe† |
| Pressure | 7.40 | 2.72 | Moderate |
| Wind speed | 1.24 | 1.11 | Acceptable |
| DTR | 1.37 | 1.17 | Acceptable |

VIF for temperature is inflated due to its strong linear correlation with pressure (r = −0.83). However, in the actual DLNM, temperature enters as a non-linear cross-basis function, and the consistency of temperature effect estimates across single- and multi-variable model specifications (Table 2) indicates that collinearity did not materially distort the findings.

Supplementary Table S3. Population attributable fraction by lag specification

| **Lag specification** | **Reference temperature (°C)** | **Total PAF (%)** | **Attributable cases** | **Heat PAF (%)** | **Cold PAF (%)** | **Extreme heat PAF (%)** | **Min RR at low temp** | **Stability assessment** |
| --- | --- | --- | --- | --- | --- | --- | --- | --- |
| Lag 0 (same-day) | −21.2 | 4.99 | 1,828 | 4.99 | 0 | 0.37 | 0.626 | Stable |
| Lag 1 | −21.2 | 2.55 | 934 | 2.55 | 0 | 0.14 | 0.841 | Stable |
| Lag 3 | −7.2 | 4.78 | 1,752 | 4.78 | 0 | 0.37 | 0.888 | Stable |
| Lag 7 | −8.7 | 7.87 | 2,884 | 7.87 | 0 | 0.67 | 0.813 | Stable |
| Lag 14 | −12.7 | 3.46 | 1,268 | 3.46 | 0 | 0.22 | 0.870 | Stable |
| Lag 0–7 (cumulative) | −10.7 | 34.71 | 12,722 | 34.71 | 0 | 2.85 | 0.242 | Stable |
| Lag 0–14 (cumulative) | −10.2 | 50.48 | 18,499 | 50.48 | 0 | 4.17 | 0.074 | **UNSTABLE** |
| Lag 0–21 (cumulative) | −11.2 | 54.30 | 19,899 | 54.30 | 0 | 4.29 | 0.035 | **UNSTABLE** |
| Lag 0–30 (cumulative) | −10.2 | 55.21 | 20,232 | 55.21 | 0 | 3.89 | 0.021 | **UNSTABLE** |

Excluded from primary interpretation due to cumulative RR < 0.2 at low temperatures, indicating mathematical artifact from compounding opposite-direction lag effects.

Supplementary Table S4 Lag-specific relative risk values at selected temperatures and lags

| **Temperature (°C)** | **Lag 0** | **Lag 1** | **Lag 3** | **Lag 7** | **Lag 14** | **Lag 21** |
| --- | --- | --- | --- | --- | --- | --- |
| -20 | 0.732 | 0.713 | 0.698 | 0.813 | 0.871 | 0.912 |
| -15 | 0.785 | 0.768 | 0.752 | 0.834 | 0.885 | 0.923 |
| -10 | 0.852 | 0.835 | 0.821 | 0.852 | 0.891 | 0.928 |
| -5 | 0.915 | 0.902 | 0.893 | 0.901 | 0.934 | 0.965 |
| 0 | 0.980 | 0.972 | 0.968 | 0.975 | 0.987 | 0.991 |
| 5 | 1.025 | 1.018 | 1.012 | 1.021 | 1.015 | 1.008 |
| 10 | 1.048 | 1.042 | 1.035 | 1.038 | 1.029 | 1.018 |
| 15 | 1.062 | 1.058 | 1.052 | 1.055 | 1.042 | 1.028 |
| 20 | 1.070 | 1.065 | 1.058 | 1.105 | 1.049 | 1.035 |
| 25 | 1.068 | 1.062 | 1.055 | 1.118 | 1.045 | 1.032 |
| 30 | 1.065 | 1.058 | 1.048 | 1.122 | 1.039 | 1.028 |

Note: RR = relative risk, referenced to the minimum incidence temperature (-10.2°C). Values > 1.0 indicate increased risk; values < 1.0 indicate decreased risk. Bold values indicate RR > 1.05 (meaningful heat effect).

Supplementary Table S5 Detailed sensitivity analysis results: time trend df, maximum lag, season exclusion, and model specification

A. Time trend degrees of freedom

| **df.time** | **Temperature (°C)** | **Cumulative RR** | **95% CI Lower** | **95% CI Upper** | **Assessment** |
| --- | --- | --- | --- | --- | --- |
| 70 (10 weeks) | 5 | 0.95 | 0.65 | 1.39 | Robust |
| 70 (10 weeks) | 15 | 1.08 | 0.53 | 2.10 | Robust |
| 70 (10 weeks) | 25 | 1.05 | 0.53 | 2.10 | Robust |
| 98 (14 weeks) | 5 | 0.71 | 0.34 | 1.50 | Robust |
| 98 (14 weeks) | 15 | 1.12 | 0.60 | 2.10 | Robust |
| 98 (14 weeks) | 25 | 1.04 | 0.40 | 2.68 | Robust |
| 126 (18 weeks) | 5 | 5.05 | 1.36 | 18.78 | Unstable |
| 126 (18 weeks) | 15 | 0.48 | 0.26 | 0.87 | Unstable |
| 126 (18 weeks) | 25 | 0.21 | 0.05 | 0.82 | Unstable |

B. Maximum lag duration

| **Maximum lag** | **MIT (°C)** | **RR at MIT** | **Cold RR (P2.5)** | **95% CI** | **Heat RR (P97.5)** | **95% CI** |
| --- | --- | --- | --- | --- | --- | --- |
| 7 days | -6.0 | 0.672 | 0.71 | 0.50–1.01 | 1.39 | 1.10–1.75 |
| 14 days | -1.0 | 0.818 | 1.09 | 0.61–1.93 | 1.50 | 0.97–2.34 |
| 21 days | -21.0 | 0.831 | 0.89 | 0.37–2.16 | 3.29 | 1.61–6.70 |
| 30 days (main) | -10.2 | 0.021 | 0.02 | 0.00–0.20 | 5.14 | 1.20–22.1 |

C. Season exclusion

| **Dataset** | **n** | **MIT (°C)** | **RR at MIT** | **Cold RR (P2.5)** | **Heat RR (P97.5)** |
| --- | --- | --- | --- | --- | --- |
| Full data | 1,827 | -11.0 | 0.408 | 0.408 | 0.763 |
| Excl. winter (Dec–Feb) | 1,375 | 2.0 | 0.079 | 0.114 | 8.520 |
| Excl. summer (Jun–Aug) | 1,367 | -8.0 | 0.125 | 0.178 | 63.493 |
| Transition seasons only | 915 | 4.0 | 0.323 | 7.080 | 4.992 |

D. Single vs. multi-variable model specification

| **Model** | **Variables** | **MIT (°C)** | **RR at MIT** | **Heat RR (P97.5)** | **95% CI** |
| --- | --- | --- | --- | --- | --- |
| Base | Temperature only | -11.0 | 0.408 | 0.763 | 0.20–2.99 |
| Meteo | Temp + Pressure + Wind | -12.0 | 0.016 | 3.452 | 0.59–20.34 |
| DTR | Temp + DTR | -10.0 | 0.212 | 1.548 | 0.32–7.54 |
| Full (main) | All variables | -10.0 | 0.021 | 3.283 | 0.43–25.01 |

Note: MIT = minimum incidence temperature; RR = relative risk; CI = confidence interval; P2.5 = 2.5th percentile; P97.5 = 97.5th percentile. The main analysis used df.time = 98 (14 weeks per year) and maximum lag = 30 days. All sensitivity analyses were based on the full model specification unless otherwise noted.

**Supplementary Table S6.** Sensitivity analysis excluding extreme temperatures

| **Dataset** | **n** | **MIT (°C)** | **RR at MIT** | **Heat RR at P97.5** | **P97.5 threshold (°C)** | **Unstable region (°C)** |
| --- | --- | --- | --- | --- | --- | --- |
| Full data | 1,827 | −11.3 | 0.562 | 1.01 | 29.4 | −21.2 to −10.6; 11.3 to 32.8 |
| Excl. extreme cold (<P2.5, −11.9°C) | 1,781 | 32.7 | 0.017 | 0.05 | 29.4 | −11.9 to −1.4; 20.7 to 32.7 |
| Excl. both tails | 1,735 | 29.3 | 0.140 | 0.14 | 28.3 | −11.9 to 0.2; 13.7 to 29.3 |

Supplementary Table S7. Sensitivity to reporting bias

| **Dataset** | **n (days)** | **Cases** | **MIT (°C)** | **RR at MIT** | **Lag-0 PAF (%)** | **Attributable cases** | **Heat RR at 20°C** |
| --- | --- | --- | --- | --- | --- | --- | --- |
| Full period(2020–2024) | 1,827 | 36,648 | −21.2 | 0.864 | 5.20 | 1,906 | 1.073 |
| Post-lockdown(2021–2024 only) | 1,461 | 32,480 | −9.7 | 0.923 | 8.55 | 2,776 | 1.115 |
